# Identification of a miRNA–mRNA regulatory network for post-stroke depression: a machine-learning approach

**DOI:** 10.3389/fneur.2023.1096911

**Published:** 2023-07-17

**Authors:** Huaide Qiu, Likui Shen, Ying Shen, Yiming Mao

**Affiliations:** ^1^Faculty of Rehabilitation Science, Nanjing Normal University of Special Education, Nanjing, China; ^2^Department of Neurosurgery, Suzhou Kowloon Hospital, Shanghai Jiao Tong University School of Medicine, Suzhou, Jiangsu, China; ^3^Rehabilitation Medicine Center, The First Affiliated Hospital of Nanjing Medical University, Nanjing, China; ^4^Department of Thoracic Surgery, Suzhou Kowloon Hospital, Shanghai Jiao Tong University School of Medicine, Suzhou, Jiangsu, China

**Keywords:** post-stroke depression, gene signatures, miRNA–mRNA network, machine-learning, diagnostics-clinical characteristic

## Abstract

**Objective:**

The study aimed to explore the miRNA and mRNA biomarkers in post-stroke depression (PSD) and to develop a miRNA–mRNA regulatory network to reveal its potential pathogenesis.

**Methods:**

The transcriptomic expression profile was obtained from the GEO database using the accession numbers GSE117064 (miRNAs, stroke vs. control) and GSE76826 [mRNAs, late-onset major depressive disorder (MDD) vs. control]. Differentially expressed miRNAs (DE-miRNAs) were identified in blood samples collected from stroke patients vs. control using the Linear Models for Microarray Data (LIMMA) package, while the weighted correlation network analysis (WGCNA) revealed co-expressed gene modules correlated with the subject group. The intersection between DE-miRNAs and miRNAs identified by WGCNA was defined as stroke-related miRNAs, whose target mRNAs were stroke-related genes with the prediction based on three databases (miRDB, miRTarBase, and TargetScan). Using the GSE76826 dataset, the differentially expressed genes (DEGs) were identified. Overlapped DEGs between stroke-related genes and DEGs in late-onset MDD were retrieved, and these were potential mRNA biomarkers in PSD. With the overlapped DEGs, three machine-learning methods were employed to identify gene signatures for PSD, which were established with the intersection of gene sets identified by each algorithm. Based on the gene signatures, the upstream miRNAs were predicted, and a miRNA–mRNA network was constructed.

**Results:**

Using the GSE117064 dataset, we retrieved a total of 667 DE-miRNAs, which included 420 upregulated and 247 downregulated ones. Meanwhile, WGCNA identified two modules (blue and brown) that were significantly correlated with the subject group. A total of 117 stroke-related miRNAs were identified with the intersection of DE-miRNAs and WGCNA-related ones. Based on the miRNA-mRNA databases, we identified a list of 2,387 stroke-related genes, among which 99 DEGs in MDD were also embedded. Based on the 99 overlapped DEGs, we identified three gene signatures (SPATA2, ZNF208, and YTHDC1) using three machine-learning classifiers. Predictions of the three mRNAs highlight four miRNAs as follows: miR-6883-5p, miR-6873-3p, miR-4776-3p, and miR-6738-3p. Subsequently, a miRNA–mRNA network was developed.

**Conclusion:**

The study highlighted gene signatures for PSD with three genes (SPATA2, ZNF208, and YTHDC1) and four upstream miRNAs (miR-6883-5p, miR-6873-3p, miR-4776-3p, and miR-6738-3p). These biomarkers could further our understanding of the pathogenesis of PSD.

## Graphical Abstract

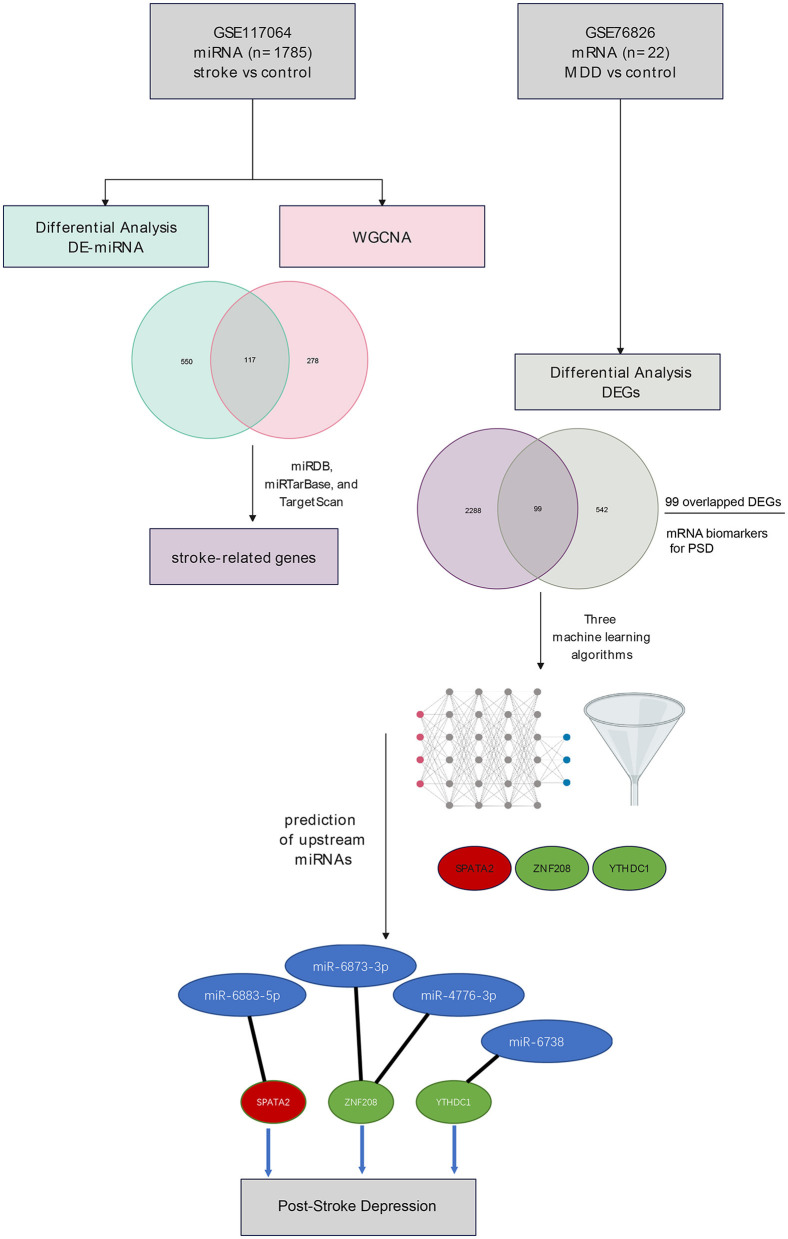

## 1. Introduction

Post-stroke depression (PSD) is a common complication after a stroke, affecting up to one-third of stroke survivors (1). It compromises individual functional recovery, impairs the quality of life, and increases the burden on the healthcare system (2). It was observed that depressive symptoms were negatively correlated with functional recovery (3) and related to higher mortality (4). However, the pathogenesis of PSD remains elusive to date.

MicroRNAs (miRNAs) are a group of small non-coding RNAs with downregulative activity on post-transcriptional gene expression by binding to the 3′ untranslated regions of target mRNAs (5, 6). Profiles of miRNA expression could be as useful as mRNA in diagnosis and prognosis (7). Of note, miRNAs can be secreted into body fluids, including peripheral blood and urine, which can be non-invasively accessed for detection (8). As such, serum miRNAs have been widely studied in various diseases, including stroke as potential biomarkers (9–12). With the development of RNA sequencing technology, data sharing, and machine-learning methods, the identification of feature serum miRNAs and the construction of related signatures in a large number of subjects have become practical. According to previous studies, serum miRNA-based signatures could effectively predict the risk of strokes in healthy individuals (13) and clinical outcomes (14, 15) in patients with neurological tumors. Moreover, miRNAs are functionally involved in numerous biological processes, including cellular metabolism (16), cell-cycle regulation (17), and immune response (18). Therefore, we hypothesized that miRNAs and their target mRNAs could be potential biomarkers implicated in the pathogenesis of PSD.

With the advancement of the machine-learning methods, the identification of relevant biomarkers becomes practical in the big data era. The least absolute shrinkage and selection operator (LASSO), a regression analysis algorithm, uses regularization to improve prediction accuracy (19). The support vector machine (SVM) is a supervised machine-learning technique widely utilized for both classification and regression (20). Random forest (RF) is considered the most accepted group classification technique because of having excellent features such as variable importance measure and out-of-bag error (21). In this study, we aimed to explore the miRNA as well as mRNA biomarkers in PSD and to construct a miRNA–mRNA regulatory network to reveal its potential pathogenesis using a machine-learning approach.

## 2. Materials and methods

### 2.1. Data source

The miRNAs expression profile was obtained from the GEO database (https://www.ncbi.nlm.nih.gov/geo/) using the accession number GSE117064, while mRNA expressions of blood samples from patients with late-onset MDD and controls were embedded in GSE76826. A total of 1,785 serum samples were incorporated into the dataset, which consists of 173 samples of patients with stroke and 1,612 controls. Extraction, detection, and data processing of serum miRNAs were provided in the previous report (13). Stroke was diagnosed based on physical and neurological examinations supplemented with brain imaging data, including computed tomography and/or magnetic resonance imaging, while healthy controls were defined as having no history of stroke and negative on medical checkup in a clinic (13). In GSE76826, there were 12 blood samples of late-onset MDD and 10 samples of controls. Late-onset MDD was defined according to the DSM-IV diagnosis and age of ≥ 50 years (22).

### 2.2. Differential analysis, WGCNA, and identification of stroke-related genes

The linear models for microarray data (LIMMA) package (23) in R software was applied to extract differentially expressed miRNAs (DE-miRNAs) between stroke and control samples. The *p*-value was adjusted with the false discovery rate (FDR) (24). An FDR of <0.05 and |*FC*| of > 1 were set as the threshold for DE-miRNAs. The visualization of differential analysis was presented with a heatmap and a volcano plot. Using GSE117064, the weighted gene correlation network analysis (WGCNA) (25, 26) was performed to construct a co-expression network to identify hub miRNA modules using the “WGCNA” package. Filtered miRNAs were employed to construct a scale-free network by calculating the connection strength between miRNAs. We assessed the correlation among miRNA modules as well as their correlations to the clinical group (stroke vs. control). Subsequently, DE-miRNAs incorporated in the stroke-related modules were identified as stroke-related miRNAs, and their target genes were predicted using miRDB (https://mirdb.org/), miRTarBase (https://mirtarbase.cuhk.edu.cn/~miRTarBase/miRTarBase_2022/php/index.php), and TargetScan (https://www.targetscan.org/vert_80/).

### 2.3. Identification of overlapped DEGs in post-stroke depression

Overlapped DEGs for post-stroke depression were defined as aberrantly expressed mRNAs in the blood sample collected from stroke patients as well as late-onset MDD. In other words, these overlapped DEGs were implicated in two diseases simultaneously. Differentially expressed mRNAs (DE-mRNAs) for late-onset MDD were defined in a similar manner with an FDR of <0.05 and |*FC*| of > 0.5. Among these DE-mRNAs, stroke-related genes were selected to identify overlapped DEGs for post-stroke depression. With these overlapped DEGs, the *clusterProfiler* R package (27) was utilized to perform the gene ontology (GO) terms and the Kyoto Encyclopedia of Genes and Genomes (KEGG) pathway enrichment analysis on the predicted target genes. Three categories were included in the GO enrichment analysis, i.e., biological process (BP), cellular component (CC), and molecular function (MF).

### 2.4. Gene signatures and a miRNA–mRNA regulatory network for PSD

The least absolute shrinkage and selection operator (LASSO), random forest (RF), and support vector machine (SVM) algorithms were utilized to build gene signatures for PSD using the overlapped DEGs. Gene signatures were established with the intersection of gene sets identified by each algorithm. The receiver operating characteristic (ROC) curves were mapped for the identified gene signatures, where the area under curves (AUCs) was calculated as an indicator of classification. The AUCs of > 0.8 were considered excellent classification, while AUCs of > 0.7 were considered acceptable. By matching miRNA–mRNA pairs in multiple databases (miRDB, miRTarBase, and TargetScan), the upstream miRNAs were predicted using the gene signatures for PSD. Subsequently, a potential miRNA–mRNA regulatory network for PSD was established.

## 3. Results

### 3.1. Differential analysis, WGCNA, and identification of stroke-related genes

**Figure 1:**
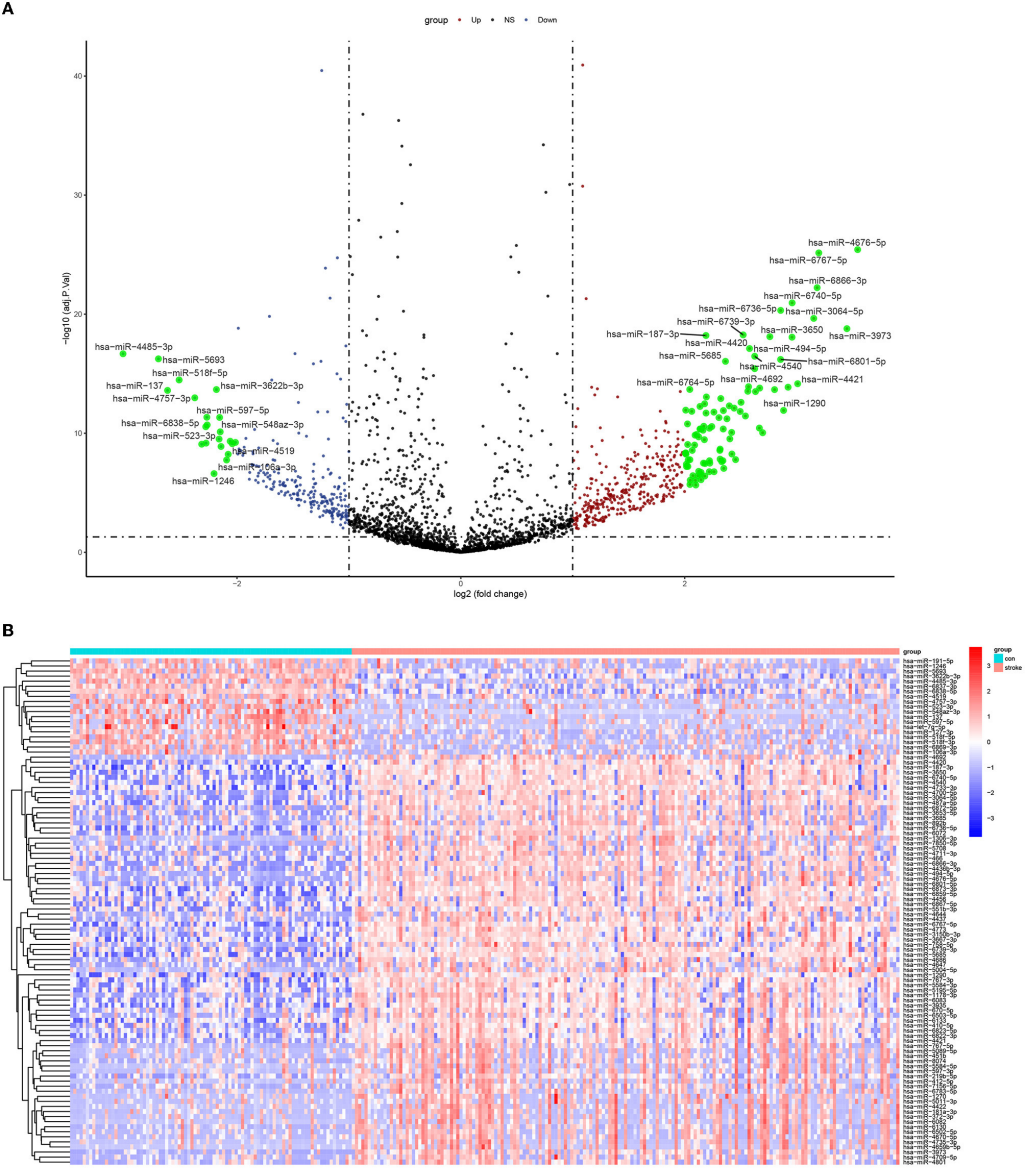
Differential analysis of miRNA expression profiles in stroke vs. control: **(A)** a volcano plot and **(B)** a heatmap with top 100 DE-miRNAs.

The overall analysis of the study was presented in the Graphical abstract. Using the LIMMA package, we retrieved a total of 667 DE-miRNAs, which included 420 upregulated and 247 downregulated ones. The feature DE-miRNAs with the absolute value of logFC of >2 were marked in green in the volcano plot (Figure 1A). Meanwhile, a heatmap showing the top 100 DE-miRNAs is presented in Figure 1B. The details of these DE-miRNAs can be found in Supplementary material 1.

**Figure 2:**
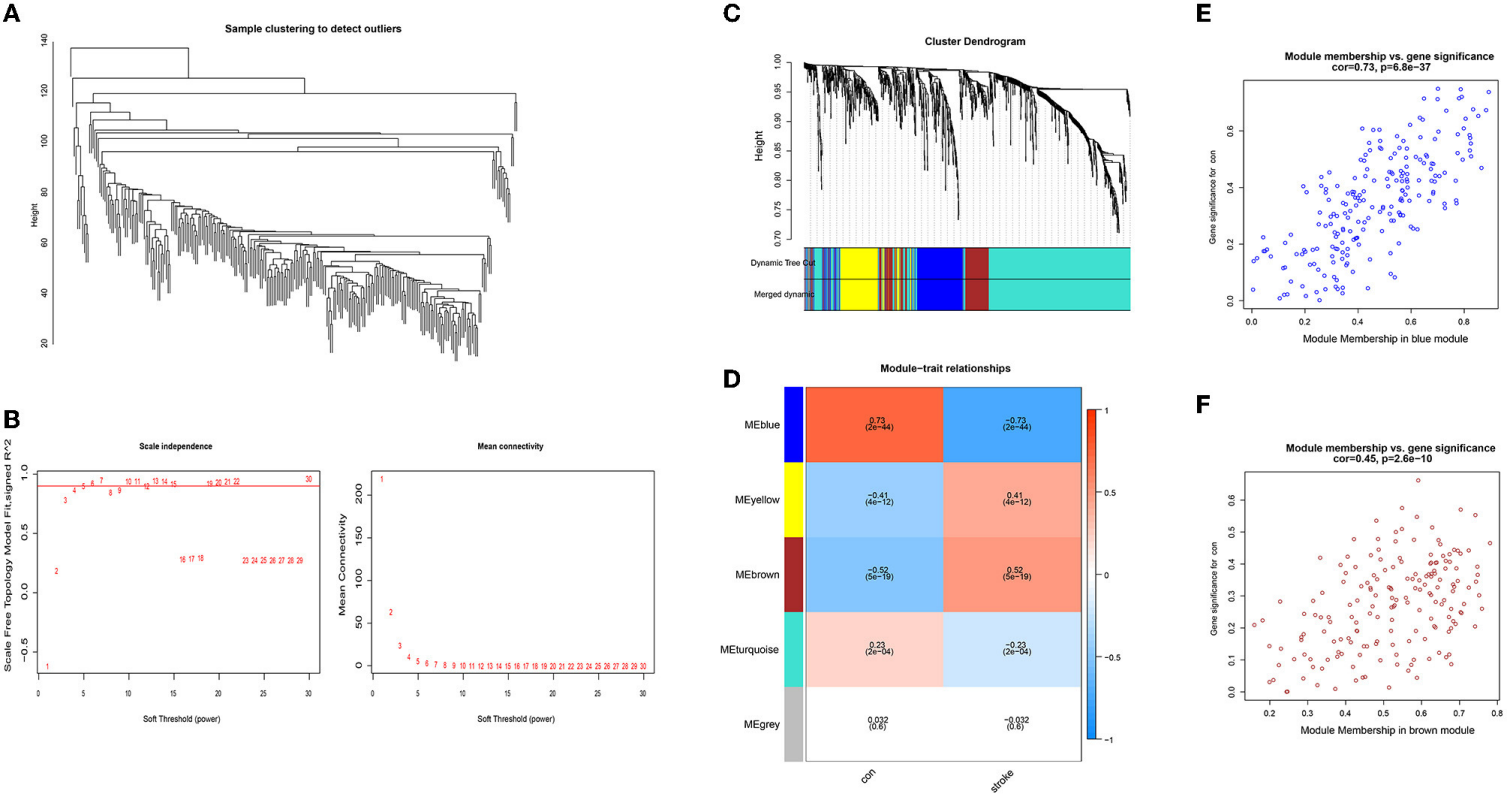
WGCNA identified two stroke-related miRNA modules: **(A)** a sample clustering tree; **(B)** soft threshold determination; **(C)** cluster dendrogram; **(D)** a module-trait correlation heatmap; **(E)** a scatter plot presenting correlation of module membership vs. gene significance in the blue module; and **(F)** a scatter plot presenting correlation of module membership vs. gene significance in the brown module.

We performed the hierarchical clustering of the samples (Figure 2A), and the soft-thresholding power was set at 5 with the cutoff score of Scale-free *R*^2^ being 0.9 (Figure 2B). The clustering dendrograms of the sample identified five modules (Figure 2C) and their correlations with clinical groups were presented in the heatmap plot (Figure 2D). Subsequently, we selected the blue and brown modules with the highest correlation coefficient for downstream analysis. The scatter plots in Figures 2E, F show a significant correlation between gene significance and module memberships in the aforementioned modules, while the details can be accessed in Supplementary material 2.

**Figure 3:**
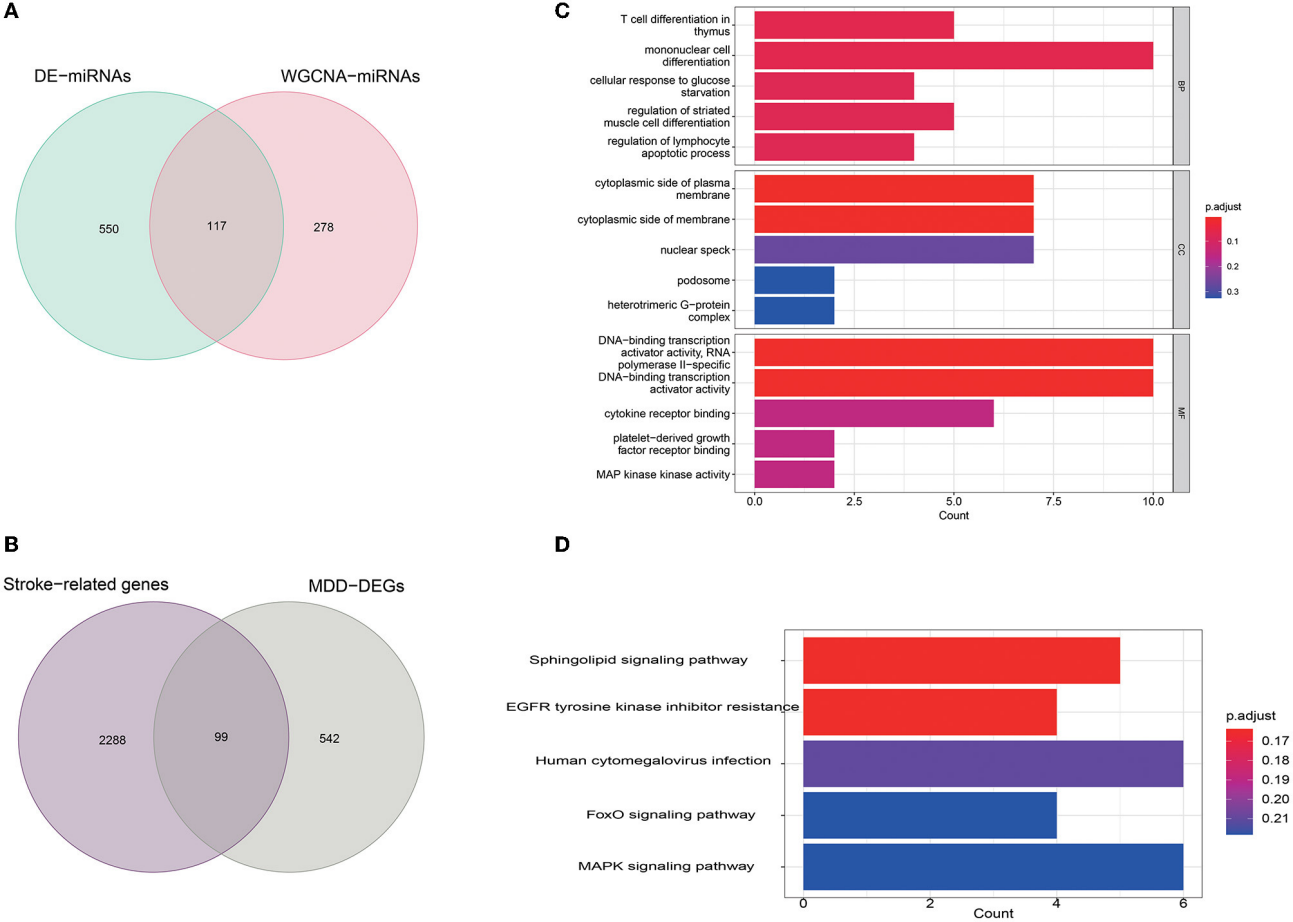
Identification of potential DEGs in PSD and enrichment analysis: **(A)** identification of stroke-related miRNAs; **(B)** identification of overlapped DEGs in PSD; **(C)** GO enrichment analysis; and **(D)** KEGG pathway analysis.

Stroke-related miRNAs were identified with the intersection of DE-miRNAs and modules of interest found in WGCNA, leading to the identification of 117 miRNAs (Figure 3A). A total of 2,387 target mRNAs were predicted with these stroke-related miRNAs according to the databases. Thus, these mRNAs were identified as stroke-related genes.

### 3.2. Identification of overlapped DEGs in post-stroke depression

Using mRNA expression profiles in GSE76826, we identified 641 DEGs with 10 samples from late-onset MDD and 12 controls. To find potential biomarkers in PSD, we intersected stroke-related genes with DEGs observed in MDD patients. As a result, 99 DEGs were identified (Figure 3B). The list of the 99 DEGs can be accessed in Supplementary material 3.

Thereafter, using *clusterProfiler* in R, GO functional and KEGG enrichment analyses were performed on the 99 DEGs to further understand their biological functions. As represented in Figure 3C, the biological process (BP) was significantly enriched in T cell differentiation in the thymus, mononuclear cell differentiation, cellular response to glucose starvation, regulation of striated muscle cell differentiation, regulation of lymphocyte apoptotic process; the cellular component (CC) was particularly enriched in the cytoplasmic side of the plasma membrane, cytoplasmic side of the membrane, nuclear speck, podosome, heterotrimeric G-protein complex; and molecular function (MF) was mainly enriched in DNA-binding transcription activator activity, RNA polymerase II-specific, DNA-binding transcription activator activity, cytokine receptor binding, platelet-derived growth factor receptor binding, and mitogen-activated protein (MAP) kinase activity.

Furthermore, the KEGG pathway analysis of the 99 DEGs is shown in Figure 3D. Among all the pathways enriched, the top five most significant pathways were as follows: sphingolipid signaling pathway, EGFR tyrosine kinase inhibitor resistance, human cytomegalovirus infection, FoxO signaling pathway, and MAPK signaling pathway. Among them, MAPK signaling pathways have been associated with the pathophysiology of PSD in several studies (28–30).

**Figure 4:**
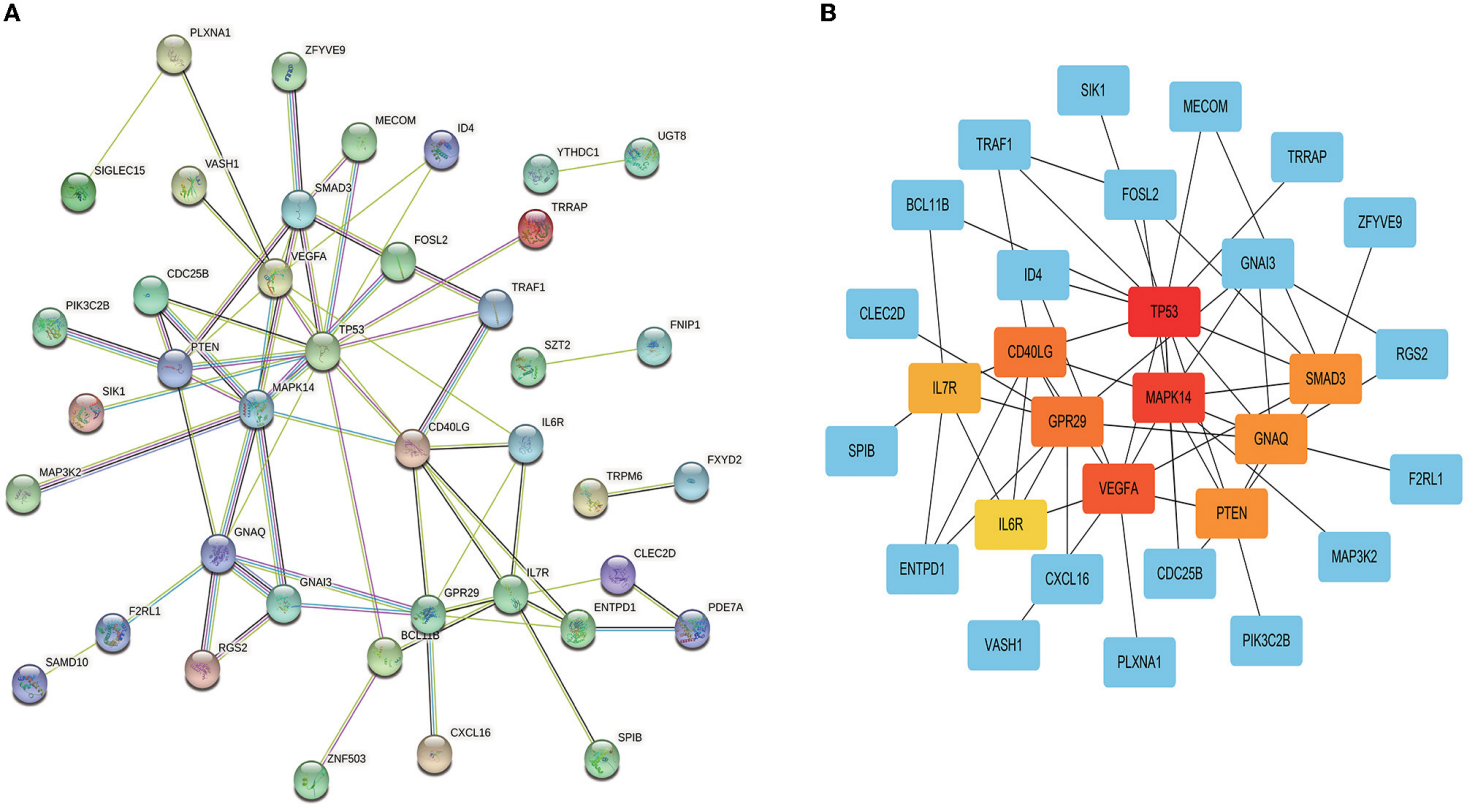
Protein–protein interaction (PPI) network using the overlapped DEGs. **(A)** PPI network based on the STRING database; **(B)** representation of the network using Cytoscape.

The PPI network of the 99 DEGs was constructed by the STRING online database with high confidence of >0.4 applied. The disconnected nodes (genes) were removed from the PPI network (Figure 4A). The network was then presented using the cytoHubba tool in Cytoscape (Figure 4B); the top 10 hub genes were as follows: TP53, MAPK14, VEGFA, GPR29, CD40LG, SMAD3, GNAQ, PTEN, IL7R, and IL6R.

### 3.3. Selection of feature mRNA by machine-learning algorithms and construction of a miRNA–mRNA network

**Figure 5:**
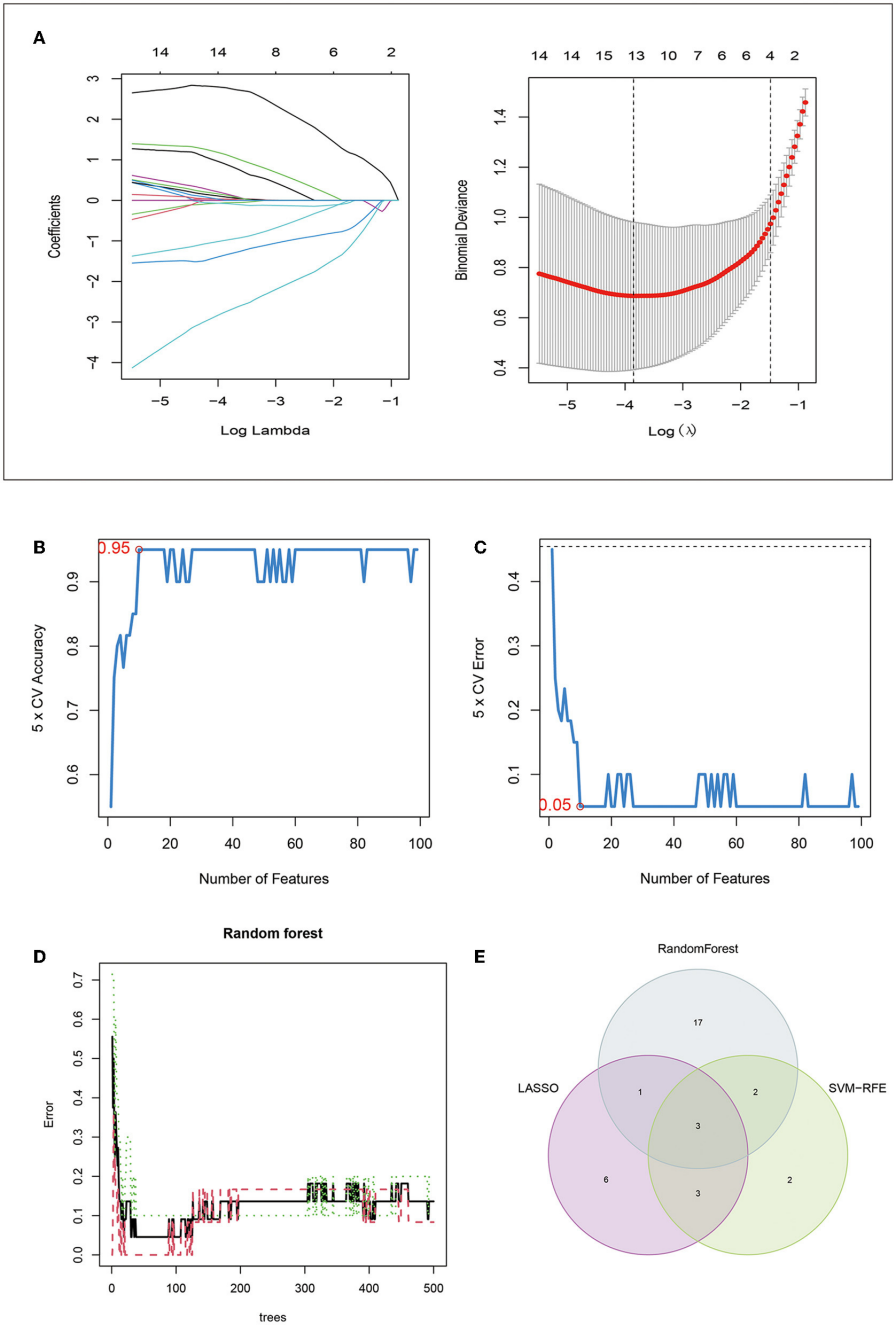
Machine-learning methods identified three hub genes in PSD: **(A)** 10-fold cross-validation for tuning parameter selection in the LASSO model, where LASSO identified 13 mRNAs; **(B)** accuracy of the SVM algorithm; **(C)** error of the estimate generation for the SVM algorithm; **(D)** relationship between model error rate and number of trees for the RF algorithm; and **(E)** feature selection with the intersection of results from LASSO, SVM, and RF algorithms.

For a further selection of the mRNA features for post-stroke depression, we used the LASSO algorithm to identify a set of 13 mRNAs (Figure 5A), the SVM algorithm to select a set of 10 mRNAs (Figures 5B, C), and the RF algorithm to select a set of 23 mRNAs (Figure 5D). Specifically, a total of 99 genes were selected by the SVM algorithm to construct the classification model using a 10-fold cross-validation. When 10 genes were selected, the accuracy of the model was the highest (Figures 5B, C). The RF algorithm screened out a total of 99 genes, and the top 23 genes with positive values of importance were selected (Supplementary material 4).

**Figure 6:**
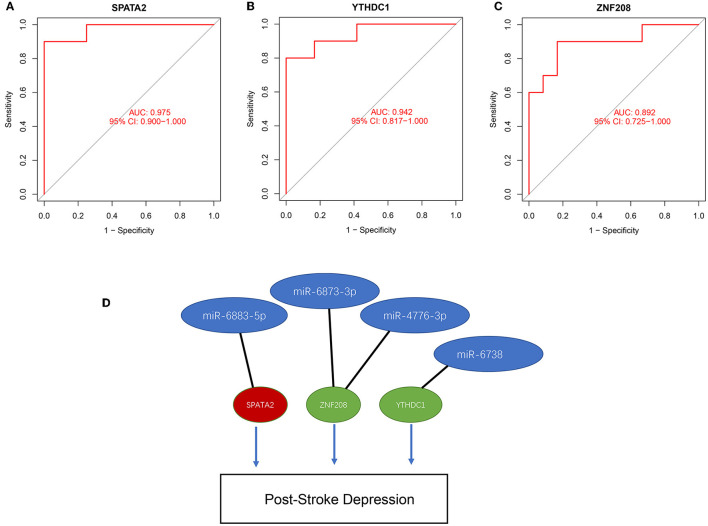
ROC validation of three hub genes **(A)** SPATA2, **(B)** YTHDC1, **(C)** ZNF208 and construction of a miRNA–mRNA network **(D)**.

RF: The RF algorithm screened out a total of 99 genes, and the top 23 genes with positive values of importance were selected (datasheet attached). After combining the mRNAs screened out via the LASSO, SVM, and RF algorithms, three diagnostic mRNAs (SPATA2, ZNF208, and YTHDC1) were identified for post-stroke depression (Figure 5E). These genes were also validated using the ROC curves with AUCs > 0.89 (Figures 6A–C). The prediction of these genes revealed a panel of four miRNAs, with which the genes form a miRNA–mRNA regulatory network, as presented in Figure 6D.

## 4. Discussion

In the present study, we identified stroke-related genes through differential analysis, WGCNA, as well as target prediction via miRNAs. Subsequently, 99 overlapped DEGs were identified in late-onset MDD to reveal potential biomarkers in PSD. Enrichment analysis revealed that these genes were implicated in pathways related to sphingolipid signaling, EGFR tyrosine kinase inhibitor resistance, human cytomegalovirus infection, FoxO signaling, and MAPK signaling. Furthermore, three machine-learning algorithms were employed to explore gene signatures for PSD, which was validated with the ROC curves. At last, a miRNA–mRNA network was constructed.

Our study highlighted gene signatures for PSD with three genes: SPATA2, ZNF208, and YTHDC1. However, there were no reports on their role in PSD. SPATA2 enables signaling receptor complex adaptor activity and ubiquitin-specific protease binding activity (31). SPATA2 is involved in several processes, including protein deubiquitination, necroptotic process, and tumor necrosis factor-mediated signaling pathway (32, 33). The knockdown of SPATA2 leads to the activation of P38MAPK and NLRP3 inflammasome and the enhancement of NF-κB signaling, indicating that SPATA2 plays a protective role against brain inflammation induced by ischemia/reperfusion injury (34). ZNF208 polymorphisms were observed to be associated with ischemic stroke in a Chinese Han population (35); however, no other reports concerning its role in stroke or depression were reported. An increasing number of studies have shown that YTHDC1, an important N6-methyladenosine (m6A) reader, plays a key role in multiple biological functions as well as in disease progression. It was observed that YTHDC1 could be protective against ischemic stroke by enhancing Akt phosphorylation via destabilizing PTEN mRNA (36). Therefore, it could be a potential therapeutic target for ischemic stroke. With the gene signatures for PSD, we predicted a list of four miRNAs (miR-6883-5p, miR-6873-3p, miR-4776-3p, and miR-6738-3p) based on the databases. Subsequently, a miRNA–mRNA network was developed, and it could shed light on the pathogenesis of PSD.

Emerging studies have investigated the role of miRNA–mRNA networks in the pathogenesis and progression of diseases, such as HBV-related hepatocellular carcinoma (37), stroke due to atrial fibrillation (38), and MDD in ovarian cancer patients (39). These studies revealed potential mechanisms by which the existing risk factor or disease contributes to the development of specific complications. In the case of PSD, datasets were exploited to find overlapped DEGs in stroke and late-onset MDD. Using three machine-learning classifiers, we further selected three feature genes and four upstream miRNAs, which could be potential targets for PSD treatment. To the best of our knowledge, the present study was the first to depict a miRNA–mRNA network for PSD; further investigations with a focus on the biological functions of these miRNAs and mRNAs are necessary. Our study suffered from a lack of wet lab validation, which may be the major limitation. However, the role of the miRNAs and mRNAs on PSD was first reported in our study, which could be of interest to further studies.

## 5. Conclusion

Our study highlighted gene signatures for PSD with three genes: SPATA2, ZNF208, and YTHDC1; their upstream miRNAs were predicted as follows: miR-6883-5p, miR-6873-3p, miR-4776-3p, and miR-6738-3p. The miRNA–mRNA network was constructed, and these biomarkers could further our understanding of the pathogenesis of PSD.

## Data availability statement

The original contributions presented in the study are included in the article/Supplementary material, further inquiries can be directed to the corresponding authors.

## Ethics statement

Ethical review and approval was not required for the study on human participants in accordance with the local legislation and institutional requirements. Written informed consent from the patients/participants or patients/participants' legal guardian/next of kin was not required to participate in this study in accordance with the national legislation and the institutional requirements.

## Author contributions

HQ, YM, and YS: data curation, formal analysis, roles/writing—original draft, and writing—review and editing. LS: data curation. YM and YS: conceptualization, design, and methodology. All authors contributed to the article and approved the submitted version.

## Supplementary material

Supplementary material 1 DE-miRNA list retrieved from GSE117064.

Supplementary material 2 Correlations between gene significance and module membership.

Supplementary material 3 List of 99 DEGs implicated in PSD.

Supplementary material 4 Gene list generated by the RF algorithm.

## References

[1] Villa RF Ferrari F Moretti A. Post-stroke depression: mechanisms and pharmacological treatment. Pharmacol Ther. (2018) 184:131–44. 10.1016/j.pharmthera.2017.11.005 29128343

[2] Guo J Wang J Sun W Liu X. The advances of post-stroke depression: 2021 update. J Neurol. (2022) 269:1236–49. 10.1007/s00415-021-10597-4 34052887

[3] Herrmann N Black SE Lawrence J Szekely C Szalai JP. The Sunnybrook Stroke Study: a prospective study of depressive symptoms and functional outcome. Stroke. (1998) 29:618–24. 10.1161/01.STR.29.3.618 9506602

[4] Ayerbe L Ayis S Crichton SL Rudd AG Wolfe CD. Explanatory factors for the increased mortality of stroke patients with depression. Neurology. (2014) 83:2007–12. 10.1212/WNL.0000000000001029 25355829 PMC4248453

[5] Ambros V. The functions of animal microRNAs. Nature. (2004) 431:350–5. 10.1038/nature02871 15372042

[6] Eulalio A Huntzinger E Izaurralde E. Getting to the root of miRNA-mediated gene silencing. Cell. (2008) 132:9–14. 10.1016/j.cell.2007.12.024 18191211

[7] Liu DZ Tian Y Ander BP Xu H Stamova BS Zhan X. Brain and blood microRNA expression profiling of ischemic stroke, intracerebral hemorrhage, and kainate seizures. J Cerebr Blood Flow Metabol. (2010) 30:92–101. 10.1038/jcbfm.2009.186 19724284 PMC2949089

[8] Matsuzaki J Ochiya T. Circulating microRNAs and extracellular vesicles as potential cancer biomarkers: a systematic review. Int J Clin Oncol. (2017) 22:413–20. 10.1007/s10147-017-1104-3 28243946

[9] do Amaral AE Rode MP Cisilotto J da Silva TE Fischer J Matiollo C. MicroRNA profiles in serum samples from patients with stable cirrhosis and miRNA-21 as a predictor of transplant-free survival. Pharmacol Res. (2018) 134:179–92. 10.1016/j.phrs.2018.06.019 29935272

[10] Dieckmann KP Radtke A Spiekermann M Balks T Matthies C Becker P. Serum levels of microRNA miR-371a-3p: a sensitive and specific new biomarker for germ cell tumours. Eur Urol. (2017) 71:213–20. 10.1016/j.eururo.2016.07.029 27495845

[11] Ji D Qiao M Yao Y Li M Chen H Dong Q. Serum-based microRNA signature predicts relapse and therapeutic outcome of adjuvant chemotherapy in colorectal cancer patients. EBioMedicine. (2018) 35:189–97. 10.1016/j.ebiom.2018.08.042 30166271 PMC6156709

[12] Rink C Khanna S. MicroRNA in ischemic stroke etiology and pathology. Physiol Genom. (2011) 43:521–8. 10.1152/physiolgenomics.00158.2010 20841499 PMC3110894

[13] Sonoda T Matsuzaki J Yamamoto Y Sakurai T Aoki Y Takizawa S. Serum MicroRNA-based risk prediction for stroke. Stroke. (2019) 50:1510–8. 10.1161/STROKEAHA.118.023648 31136284

[14] Zhi F Shao N Wang R Deng D Xue L Wang Q. Identification of 9 serum microRNAs as potential noninvasive biomarkers of human astrocytoma. Neuro Oncol. (2015) 17:383–91. 10.1093/neuonc/nou169 25140035 PMC4483096

[15] Zhao H Shen J Hodges TR Song R Fuller GN Heimberger AB. Serum microRNA profiling in patients with glioblastoma: a survival analysis. Mol Cancer. (2017) 16:59. 10.1186/s12943-017-0628-5 28284220 PMC5346242

[16] Ouimet M Ediriweera HN Gundra UM Sheedy FJ Ramkhelawon B Hutchison SB. MicroRNA-33–dependent regulation of macrophage metabolism directs immune cell polarization in atherosclerosis. J Clin Invest. (2015) 125:4334–48. 10.1172/JCI81676 26517695 PMC4665799

[17] Bueno MJ Malumbres M. MicroRNAs and the cell cycle. Biochim Biophys Acta. (2011) 1812:592–601. 10.1016/j.bbadis.2011.02.002 21315819

[18] Saba R Sorensen DL Booth SA. MicroRNA-146a: a dominant, negative regulator of the innate immune response. Front Immunol. (2014) 5:578. 10.3389/fimmu.2014.00578 25484882 PMC4240164

[19] Ranstam J Cook J. LASSO regression. J Br Surg. (2018) 105:1348. 10.1002/bjs.10895

[20] Mahesh B. Machine learning algorithms-a review. Int J Sci Res. (2020) 9:381–6. 10.21275/ART20203995

[21] Abdulkareem NM Abdulazeez AM. Machine learning classification based on Radom Forest Algorithm: a review. Int J Sci Bus. (2021) 5:128–42. 10.5281/zenodo.4471118

[22] Miyata S Kurachi M Okano Y Sakurai N Kobayashi A Harada K. Blood transcriptomic markers in patients with late-onset major depressive disorder. PLoS ONE. (2016) 11:e0150262. 10.1371/journal.pone.0150262 26926397 PMC4771207

[23] Ritchie ME Belinda P Di W Hu Y Law CW Wei S. In limma powers differential expression analyses for RNA-sequencing and microarray studies. Nucl Acids Res. (2015) 7:gkv007. 10.1093/nar/gkv007 25605792 PMC4402510

[24] Benjamini Y Hochberg Y. Controlling the false discovery rate: a practical and powerful approach to multiple testing. J Royal Stat Soc Ser B. (1995) 57:289–300. 10.1111/j.2517-6161.1995.tb02031.x

[25] Zhang B Horvath S. A general framework for weighted gene co-expression network analysis. Stat Appl Genet Mol Biol. (2005) 4:17. 10.2202/1544-6115.1128 16646834

[26] Oldham MC Konopka G Iwamoto K Langfelder P Kato T Horvath S. Functional organization of the transcriptome in human brain. Nat Neurosci. (2008) 11:1271–82. 10.1038/nn.2207 18849986 PMC2756411

[27] Yu G Wang LG Han Y He QY. clusterProfiler: an R package for comparing biological themes among gene clusters. Omics. (2012) 16:284–7. 10.1089/omi.2011.0118 22455463 PMC3339379

[28] Feng Y Li X Wang J Huang X Meng L Huang J. Pyruvate kinase M2 (PKM2) improve symptoms of post-ischemic stroke depression by activating VEGF to mediate the MAPK/ERK pathway. Brain Behav. (2022) 12:e2450. 10.1002/brb3.2450 34898024 PMC8785619

[29] Verma R Harris NM Friedler BD Crapser J Patel AR Venna V. Reversal of the detrimental effects of post-stroke social isolation by pair-housing is mediated by activation of BDNF-MAPK/ERK in aged mice. Sci Rep. (2016) 6:1–13. 10.1038/srep25176 27125783 PMC4850427

[30] Zhang Y Cheng L Chen YG Yang Y Liu J Zeng L. Clinical predictor and circulating microRNA profile expression in patients with early onset post-stroke depression. J Affect Disord. (2016) 193:51–8. 10.1016/j.jad.2015.12.061 26766035

[31] Schlicher L Brauns-Schubert P Schubert F Maurer U. SPATA2: more than a missing link. Cell Death Differ. (2017) 24:1142–7. 10.1038/cdd.2017.26 28282038 PMC5520165

[32] Elliott PR Leske D Hrdinka M Bagola K Fiil BK McLaughlin SH. SPATA2 links CYLD to LUBAC, activates CYLD, and controls LUBAC signaling. Mol Cell. (2016) 63:990–1005. 10.1016/j.molcel.2016.08.001 27591049 PMC5031558

[33] Yang XD Li W Zhang S Wu D Jiang X Tan R. PLK4 deubiquitination by Spata2-CYLD suppresses NEK7-mediated NLRP3 inflammasome activation at the centrosome. EMBO J. (2020) 39:e102201. 10.15252/embj.2019102201 31762063 PMC6960439

[34] Ren Y Jiang J Jiang W Zhou X Lu W Wang J. Spata2 knockdown exacerbates brain inflammation via NF-κB/P38MAPK signaling and NLRP3 inflammasome activation in cerebral ischemia/reperfusion rats. Neurochem Res. (2021) 46:2262–75. 10.1007/s11064-021-03360-8 34075523

[35] Yu J Zhou F Luo D Wang N Zhang C Jin T. ZNF208 polymorphisms associated with ischemic stroke in a southern Chinese Han population. J Gene Med. (2017) 19:1–2. 10.1002/jgm.2937 27936511

[36] Zhang Z Wang Q Zhao X Shao L Liu G Zheng X. YTHDC1 mitigates ischemic stroke by promoting Akt phosphorylation through destabilizing PTEN mRNA. Cell Death Dis. (2020) 11:977. 10.1038/s41419-020-03186-2 33188203 PMC7666223

[37] Lou W Liu J Ding B Chen D Xu L Ding J. Identification of potential miRNA-mRNA regulatory network contributing to pathogenesis of HBV-related HCC. J Transl Med. (2019) 17:7. 10.1186/s12967-018-1761-7 30602391 PMC6317219

[38] Zou R Zhang D Lv L Shi W Song Z Yi B. Bioinformatic gene analysis for potential biomarkers and therapeutic targets of atrial fibrillation-related stroke. J Transl Med. (2019) 17:45. 10.1186/s12967-019-1790-x 30760287 PMC6375208

[39] Wu C Zhao Y Liu Y Yang X Yan M Min Y. Identifying miRNA-mRNA regulation network of major depressive disorder in ovarian cancer patients. Oncol Lett. (2018) 16:5375–82. 10.3892/ol.2018.9243 30214617 PMC6126176

